# Distinct regions within the GluN2C subunit regulate the surface delivery of NMDA receptors

**DOI:** 10.3389/fncel.2014.00375

**Published:** 2014-11-10

**Authors:** Katarina Lichnerova, Martina Kaniakova, Kristyna Skrenkova, Ladislav Vyklicky, Martin Horak

**Affiliations:** ^1^Institute of Physiology, Academy of Sciences of the Czech Republic v.v.i., PragueCzech Republic; ^2^Department of Physiology, Faculty of Science, Charles University in PraguePrague, Czech Republic

**Keywords:** glutamate receptor, ion channel, intracellular trafficking, electrophysiology, cerebellar granule cells, endoplasmic reticulum

## Abstract

*N*-methyl-D-aspartate (NMDA) receptors mediate fast excitatory synaptic transmission in the mammalian central nervous system. The activation of NMDA receptors plays a key role in brain development, synaptic plasticity, and memory formation, and is a major contributor to many neuropsychiatric disorders. Here, we investigated the mechanisms that underlie the trafficking of GluN1/GluN2C receptors. Using an approach combining molecular biology, microscopy, and electrophysiology in mammalian cell lines and cultured cerebellar granule cells, we found that the surface delivery of GluN2C-containing receptors is reduced compared to GluN2A- and GluN2B-containing receptors. Furthermore, we identified three distinct regions within the N-terminus, M3 transmembrane domain, and C-terminus of GluN2C subunits that are required for proper intracellular processing and surface delivery of NMDA receptors. These results shed new light on the regulation of NMDA receptor trafficking, and these findings can be exploited to develop new strategies for treating some forms of neuropsychiatric disorders.

## INTRODUCTION

*N*-methyl-D-aspartate (NMDA) receptors are ionotropic glutamate receptors that play a key role in glutamatergic neurotransmission. NMDA receptors are heterotetramers composed of GluN1, GluN2, and/or GluN3 subunits. The GluN1 subunit is encoded by a single gene that expresses eight splice variants. GluN2 subunits are encoded by four different genes, giving rise to GluN2A, GluN2B, GluN2C, and GluN2D subunits; finally, GluN3 subunits are encoded by two genes, giving rise to GluN3A and GluN3B subunits (Lau and Zukin, 2007; Petralia et al., 2009; Traynelis et al., 2010). The current consensus is that functional NMDA receptors are composed primarily of two GluN1 subunits and two GluN2 subunits, and their activation requires both glutamate and the co-agonist glycine (Traynelis et al., 2010). All NMDA receptor subunits share the following structural features: (i) four membrane-spanning segments (M1–M4), which help form the channel’s pore; (ii) an extracellular N-terminus and an extracellular loop between M3 and M4; and (iii) an intracellular C-terminus (Madden, 2002; Traynelis et al., 2010).

The GluN2A through GluN2D subunits have expression patterns that vary widely both in time (i.e., during development) and in space (i.e., among various brain regions; Paoletti, 2011). For example, in cerebellar granule cells (CGCs), GluN2B subunits are expressed early in development but disappear almost entirely by postnatal day 21; in contrast, GluN2A and GluN2C subunits are expressed later in development (Akazawa et al., 1994; Monyer et al., 1994). In addition to its expression in the cerebellum, low levels of GluN2C mRNA have also been found in the hippocampus (Pollard et al., 1993). GluN2C-containing NMDA receptors have distinct functional properties, including reduced magnesium affinity and reduced conductance, and these properties are conferred upon the receptor’s synaptic currents (Lu et al., 2006; Paoletti, 2011). Interestingly, the GRIN2C gene, which encodes the GluN2C subunit, has several splice variants (Rafiki et al., 2000), and its expression is perturbed in some neurological disorders (Marianowski et al., 1995; Kadotani et al., 1998).

It is generally believed that the number and type of NMDA receptors present at the cell surface are regulated at multiple levels, including their synthesis, subunit assembly, processing in the endoplasmic reticulum (ER), intracellular trafficking, and degradation. Studies have shown that before a functional NMDA receptor heterotetramer is formed in the ER, GluN1, and GluN2 monomers form an intermediate complex, for example GluN1-GluN1 and/or GluN1-GluN2 dimers (Atlason et al., 2007; Schuler et al., 2008). These intermediate complexes likely employ specific ER retention mechanisms, as they are not trafficked from the ER (with the exception of certain GluN1 splice variants; Mcllhinney et al., 1998; Okabe et al., 1999; Fukaya et al., 2003). Although distinct regions within the GluN1, GluN2A, and GluN2B subunits regulate ER processing and the trafficking of functional NMDA receptors (Standley et al., 2000; Meddows et al., 2001; Mu et al., 2003; Hawkins et al., 2004; Horak et al., 2008; Horak and Wenthold, 2009; Kenny et al., 2009; Qiu et al., 2009), subunit-dependent differences in early NMDA receptor processing (e.g., between GluN1/GluN2A-B and GluN1/GluN2C-D receptors) have not yet been studied in detail.

Here, we determined which structural features of the GluN2C subunit regulate the surface expression of GluN2C-containing NMDA receptors. By combining microscopy and electrophysiology recordings of heterologous cells and cultured CGCs that express recombinant GluN subunits, we found that the surface expression of GluN1/GluN2C receptors is reduced compared to GluN1/GluN2A and GluN1/GluN2B receptors. Furthermore, using a panel of truncated and otherwise mutated GluN2C subunits, we identified three distinct regions in the GluN2C subunit—specifically, within the N-terminus, M3 domain, and C-terminus—that regulate the surface expression of GluN2C-containing NMDA receptors. Interestingly, trafficking of GluN1/GluN2A receptors is also regulated by the N-terminal and M3 domain–mediated mechanisms; however, the C-terminal–mediated mechanism appears to be specific to GluN2C-containing receptors. We conclude that the GluN2C subunit uses several regulatory mechanisms to control the early processing of functional NMDA receptors.

## MATERIALS AND METHODS

### MOLECULAR BIOLOGY

The following cDNAs encoding full-length or truncated NMDA receptor subunits were used: extracellular-tagged yellow fluorescent protein (YFP)-GluN1-1a and extracellular-tagged green fluorescent protein (GFP)-GluN2A, GFP-GluN2B, and GFP-GluN2C (Luo et al., 2002; Horak et al., 2008; Chen and Roche, 2009). Untagged versions of the GluN1-1a, GluN2A, and GluN2C subunits were also used (Horak et al., 2006). Point mutations were generated using the Quick-Change site-directed mutagenesis kit (Agilent Technologies, Santa Clara, CA, USA) in accordance with the manufacturer’s instructions. The amino acid residues are numbered as published (Ishii et al., 1993). All constructs were verified by DNA sequencing.

### HETEROLOGOUS CELL CULTURE

African green monkey kidney fibroblast (COS-7) cells were cultured in Minimum Essential Medium with Earle’s salts (MEM) containing 10% (v/v) fetal bovine serum (FBS; Invitrogen, Carlsbad, CA, USA). COS-7 cells were used for microscopy experiments because they remain attached to glass coverslips during extensive washing procedures. Human embryonic kidney 293 (HEK293) cells were cultured in Opti-MEM I (Invitrogen) containing 5% FBS (v/v); these cells were used for electrophysiology.

For transfection, equal amounts of the various cDNAs (0.9 μg in total) were added to 2 μl Lipofectamine 2000 (Invitrogen) in accordance with the manufacturer’s instructions, and the DNA–Lipofectamine complexes were added to COS-7 or HEK293 cells for 5 h as described previously (Horak et al., 2008). The transfected cells were cultured in Opti-MEM I containing 1% FBS (v/v) supplemented with 20 mM MgCl_2_, 1 mM DL-2-amino-5-phosphonopentanoic acid, and the NMDA receptor antagonist kynurenic acid (3 mM) to prevent cytotoxicity caused by NMDA receptor activation. All experiments were performed within 24–48 h of transfection.

### PRIMARY CEREBELLAR GRANULE CELLS

Cerebellar granule cells were prepared from postnatal day 6–8 rats as described previously (Prybylowski et al., 2005). In brief, cells were cultured in Basal Eagle’s Medium (Invitrogen) supplemented with 10% FBS (v/v), 2 mM glutamine, and 25 mM KCl. After 5 days in culture (DIV5), the CGCs were transfected using the calcium phosphate technique as described previously (Prybylowski et al., 2002). Microscopy experiments were performed within 48–72 h of transfection. All experimental procedures involving animals were performed in accordance with the guidelines of our institute’s Animal Care Committee.

### MICROSCOPY

To surface-label the NMDA receptor subunits, COS-7 cells and CGCs were washed in phosphate-buffered saline (PBS), then incubated on ice for 15 min in a blocking solution containing PBS and 10% (v/v) normal goat serum (NGS) as described previously (Horak et al., 2008). The cells were then incubated for 30 min in blocking solution containing polyclonal rabbit anti-GFP antibody (Merck Millipore, Darmstadt, Germany; 1:1000). Next, the cells were washed twice in PBS, then incubated for 30 min in blocking solution containing the following fluorescent secondary antibodies: Alexa Fluor 555 goat anti-rabbit IgG (Invitrogen) for the COS-7 cells, or Alexa Fluor 647 goat anti-rabbit IgG (Invitrogen) for the CGCs. The cells were then washed twice in PBS and fixed for 20 min in PBS containing 4% paraformaldehyde (w/v) for 20 min. The COS-7 cells were then mounted using ProLong Antifade reagent (Invitrogen). The CGCs were processed further for intracellular labeling of the total pool of NMDA receptor subunits. In brief, the cells were permeabilized for 5 min in PBS containing 0.1% Triton X-100 (w/v), blocked for 1 h with blocking solution containing 0.1% Triton X-100, and incubated in the primary (anti-GFP) and secondary (Alexa Fluor 488 goat anti-rabbit IgG; Invitrogen) antibodies for 1 h each.

For the internalization studies, live cells were washed in PBS, then incubated on ice for 30 min in the primary (anti-GFP) antibody to label the surface receptors. The cells were then washed in PBS, and the coverslips were returned to conditioned medium for 30 min at 37°C. The cells were washed in PBS, incubated in an unconjugated goat anti-rabbit antibody (Invitrogen), fixed, permeabilized, incubated with a fluorescent secondary antibody, washed, and mounted using ProLong Antifade reagent (Lavezzari et al., 2004; Scott et al., 2004). To visualize both the surface and total pools of NMDA receptors, z-stack images were scanned using an Olympus scan®; fluorescence microscope (COS-7 cells) or a Leica SPE confocal microscope (CGCs); the images were analyzed using ImageJ software (NIH, Bethesda, MD, USA). For the microscopy experiments, ≥45 transfected COS-7 cells from ≥3 independent experiments and ≥20 transfected CGCs (unless stated otherwise) were used for analysis as described previously (Horak et al., 2008). All summary data are expressed as mean ± SEM. Differences were analyzed using the unpaired Student’s *t*-test or one-way ANOVA followed by the Dunn’s test.

### ELECTROPHYSIOLOGY

Whole-cell voltage-clamp recordings were performed using an Axopatch 200B patch-clamp amplifier (Molecular Devices, Union City, CA, USA) with compensation for both capacitance and series resistance. The extracellular solution contained (in mM): 160 NaCl, 2.5 KCl, 10 4-(2-hydroxyethyl)-1-piperazineethanesulfonic acid (HEPES), 10 glucose, 0.7 CaCl_2_, 0.2 EDTA, and 10 μM glycine (pH adjusted to 7.3 with NaOH. Glass patch pipettes (3–5 MΩ tip resistance) were filled with an intracellular solution containing (in mM): 125 gluconic acid, 15 CsCl, 5 EGTA, 10 HEPES, 3 MgCl_2_, 0.5 CaCl_2_, and 2 ATP-Mg salt (pH adjusted to 7.2 with CsOH). A microprocessor-controlled multi-barrel rapid-perfusion system (the time constant of solution exchange in the vicinity of the cells was ∼20 ms) was used to apply the test solutions (Kaniakova et al., 2012a). The experiments were performed at room temperature. Glutamate-induced responses were low-pass filtered at 2 kHz with an eight-pole Bessel filter, digitally sampled at 5 kHz, and analyzed using pCLAMP version 9 (Molecular Devices). All summary data are expressed as mean ± SEM. Differences were analyzed using the unpaired Student’s *t*-test or one-way ANOVA followed by the Dunn’s test.

## RESULTS

### THE IDENTITY THE SPECIFIC GluN2 SUBUNIT TYPE DETERMINES SURFACE DELIVERY OF THE NMDA RECEPTOR

**FIGURE 1:**
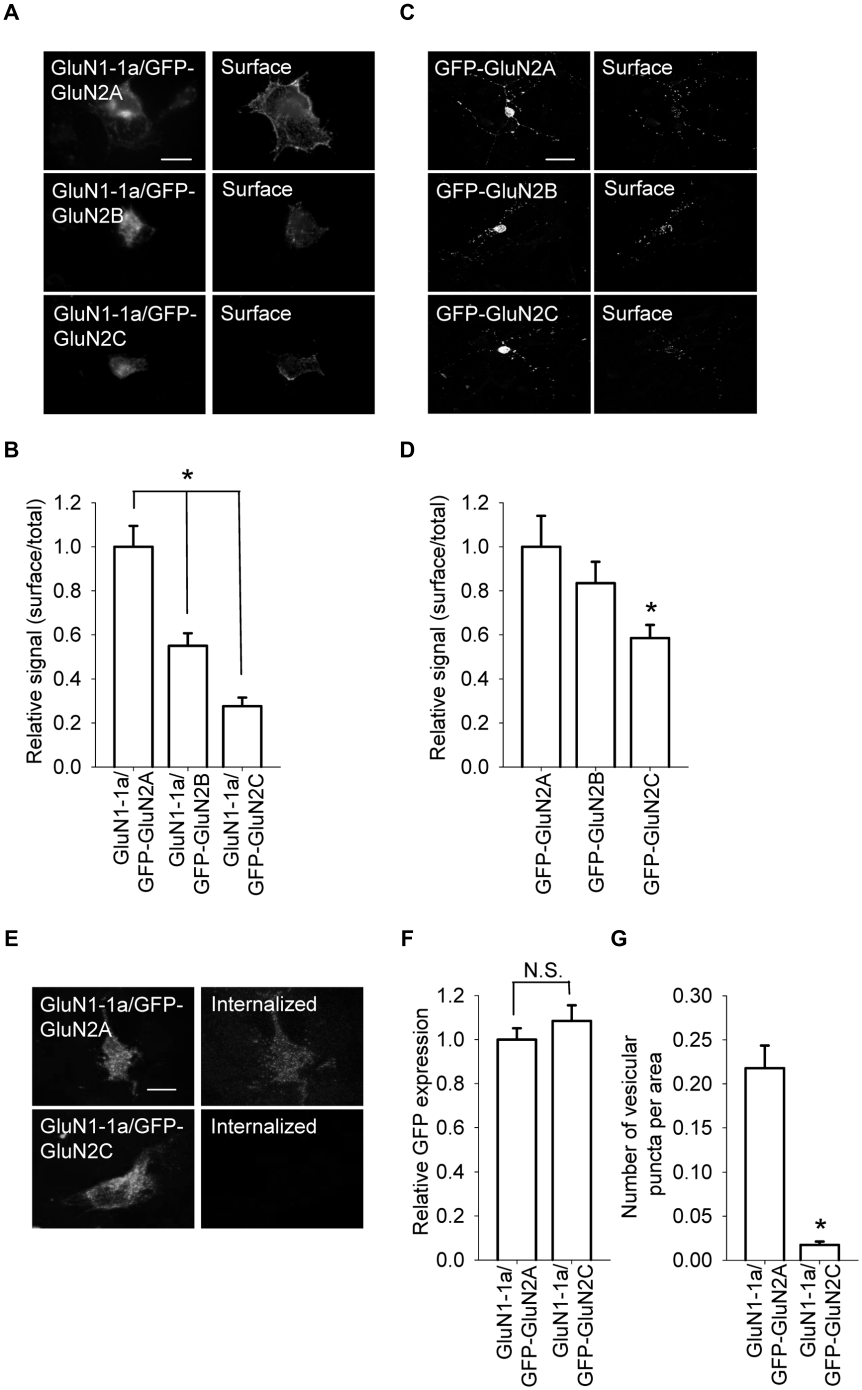
**GluN1/GluN2A, GluN1/GluN2B, and GluN1/GluN2C receptors are differentially expressed at the cell surface. (A)** Representative images of the total (left panel) and surface (right panel) pools of GluN1/GluN2A, GluN1/GluN2B, and GluN1/GluN2C receptors expressed in COS-7 cells. Scale bar, 20 μm. **(B)** Summary of the normalized intensity ratios of surface and total NMDA receptors expressed in COS-7 cells and visualized using immunofluorescence. *p < 0.05 (relative to GluN1-1a/GFP-GluN2A); ANOVA. **(C)** Representative images of total (left panel) and surface (right panel) NMDA receptor pools in cerebellar granule cells (CGCs). Scale bar, 20 μm. **(D)** Summary of the ratio of surface and total expression of NMDA receptors visualized using confocal microscopy. *p < 0.05 (relative to GluN1-1a/GFP-GluN2A); ANOVA. **(E)** Internalization of GluN1/GluN2 receptors in transfected COS-7 cells. Live cells were incubated for 30 min at 37°C with an anti-GFP antibody; the cells were then fixed and incubated with a fluorescent secondary antibody. Representative images of the GFP signal (left) and internalized receptors (right) in transfected cells are shown. Scale bar, 20 μm.** (F,G)** Summary (*n* ≥ 40 from three independent experiments) of GFP expression **(F)** and the average number of vesicular puncta per area **(G)** for the indicated NMDA receptors. *p < 0.05 (relative to GFP-GluN2A); Student’s *t*-test.

Previous studies examined the molecular mechanisms that underlie the early trafficking of GluN1/GluN2A and GluN1/GluN2B receptors; however, the trafficking of other NMDA receptor types—including GluN1/GluN2C receptors—has been largely neglected. Therefore, the aim of this study was to examine the role that various regions within the GluN2C subunit play in delivering GluN1/GluN2C receptors to the surface membrane of mammalian cell lines and cultured CGCs. We first measured the surface expression of NMDA receptors comprised of GluN1-1a together with GFP-GluN2A, GFP-GluN2B, or GFP-GluN2C subunits in transfected COS-7 cells (**Figures 1A,B**). Our data show that GluN1-1a/GFP-GluN2A receptors were expressed at the cell surface at significantly higher levels than GluN1-1a/GFP-GluN2B receptors; this finding is consistent with previous results (Chen et al., 1999). Interestingly, however, the surface expression of GluN1-1a/GFP-GluN2C receptors was lower than both GluN1-1a/GFP-GluN2A and GluN1-1a/GFP-GluN2B receptors, even though all three GluN2 subunits were expressed at similar levels (**Figures 1A,B**). Similar results were obtained when we examined the surface expression of YFP-GluN1-1a/GluN2A and YFP-GluN1-1a/GluN2C receptors in COS-7 cells (Figure S1). Finally, consistent with its strict requirement for delivering the receptor to the surface membrane, when the GluN1 subunit was not co-transfected, none of the GluN2 subunits (i.e., GFP-GluN2A, GFP-GluN2B, or GFP-GluN2C) reached the cell surface (Figure S2).

Next, we used cultured CGCs to further examine whether the surface expression of GluN2C subunits is reduced compared to GluN2A and GluN2B subunits. Cultured CGCs are an ideal model system for these experiments, as these neurons are relatively homogeneous, thus allowing us to detect relatively small changes in the surface and total expression of GluN subunits; moreover, the subunits are expressed in their native environment (Prybylowski et al., 2002; Traynelis et al., 2010). We found that compared to GluN2A and GluN2B subunits, GluN2C subunits are expressed at the surface at significantly lower levels; as with the heterologous cells, the total expression levels were similar among all three GluN2 subunits (**Figures 1C,D**). Together, these results suggest that the GluN2C subunit contains unique structural element(s) that regulate the surface delivery of NMDA receptors.

The reduced surface expression of GluN1/GluN2C receptors may be due to a faster internalization rate compared to GluN1/GluN2A and GluN1/GluN2B receptors. To test this possibility, we performed an internalization assay for GluN1-1a/GluN2A and GluN1-1a/GluN2C receptors expressed in COS-7 cells (**Figures 1E–G**). We found that GluN1-1a/GluN2C receptors internalize more slowly than GluN1-1a/GluN2A receptors; thus, the presence of the GluN2C subunit must regulate forward trafficking of the receptor rather than decreasing the receptor’s surface stability.

### DISTINCT REGIONS WITHIN THE GluN2C SUBUNIT REGULATE THE FORWARD TRAFFICKING OF NMDA RECEPTORS

**FIGURE 2:**
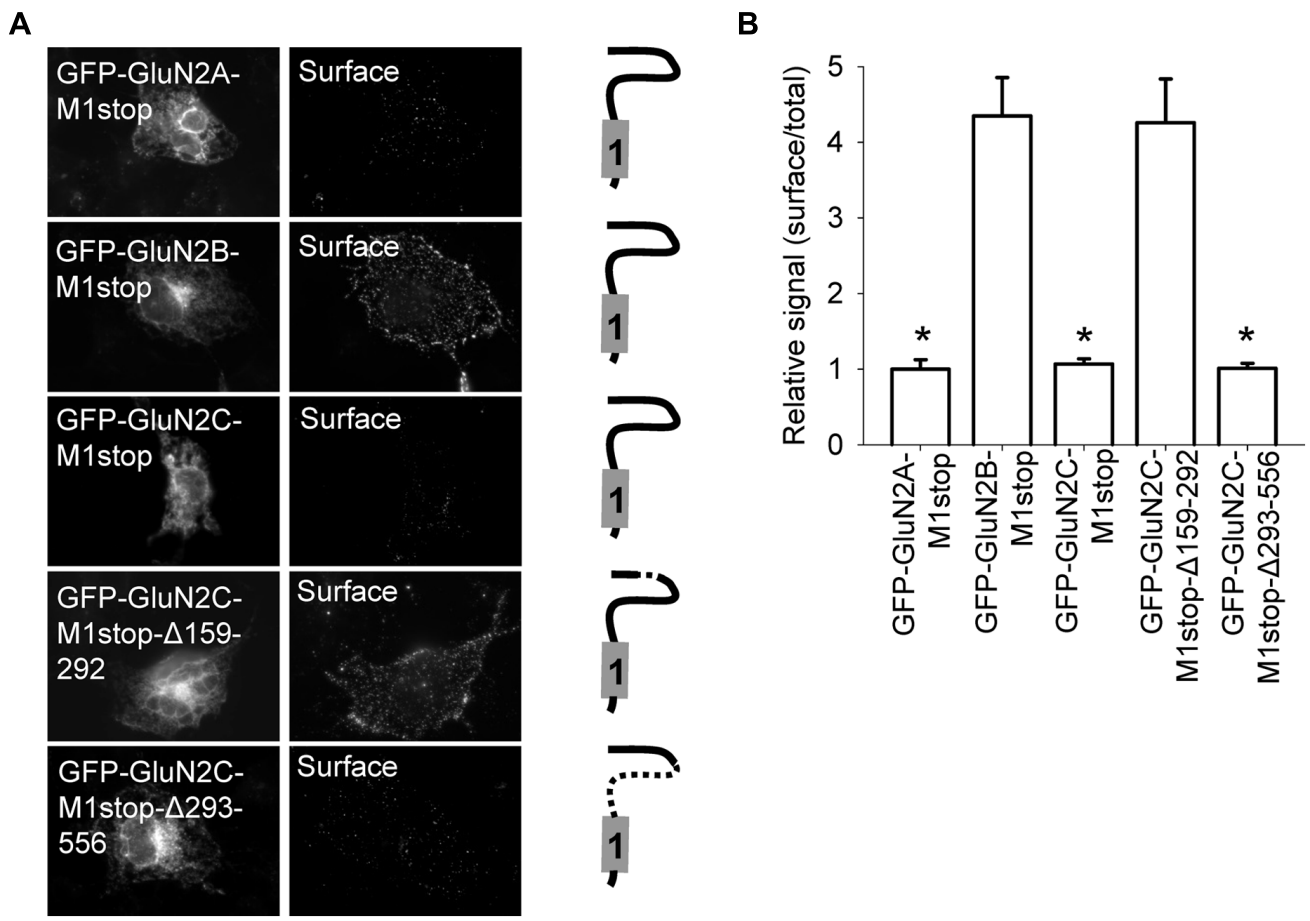
**Surface delivery of truncated GluN2 subunits. (A)** Schematic drawings of the membrane topology of the indicated truncated GluN2 subunits and representative images of total (left panel) and surface (right panel) pools of NMDA receptors expressed in COS-7 cells. **(B)** Summary of the normalized ratios of surface and total expression of the indicated NMDA receptor subunits measured using fluorescence microscopy. *p < 0.05 (relative to GFP-GluN2A-M1stop); ANOVA.

Previous studies identified several regions within NMDA receptor subunits—including the N-terminus, membrane domains, and C-terminus—as key elements for controlling the delivery of NMDA receptors to the cell surface (Stephenson et al., 2008; Traynelis et al., 2010). Moreover, the N-terminal domain of the GluN2A subunit—but not the GluN2B subunit—contains an ER retention signal (Horak et al., 2008; Qiu et al., 2009). We first confirmed that a GluN2A subunit that is truncated after the M1 domain (GFP-GluN2A-M1stop), but not the equivalent truncated GluN2B subunit (GFP-GluN2B-M1stop), is retained in the intracellular compartment (**Figures 2A,B**). We next examined the trafficking of GFP-GluN2C-M1stop and found that this truncated GluN2C protein is retained in the intracellular compartment; thus, the N-terminal domains of both GluN2A and GluN2C regulate their intracellular processing, perhaps via a similar mechanism (**Figures 2A,B**). We also generated two new GFP-GluN2C-M1stop constructs that lack either the A2 segment (GFP-GluN2C-M1stop-Δ159–292) or the region immediately downstream of the A2 segment (GFP-GluN2C-M1stop-Δ293–556). As we expected, deleting the A2 segment prevented the intracellular retention of the truncated GluN2C subunit, similar to a previous study using the GluN2A subunit (Qiu et al., 2009); moreover, the GFP-GluN2C-M1stop-Δ293–556 subunit was still retained intracellularly (**Figures 2A,B**). Using confocal microscopy, we found that GFP-GluN2A-M1stop and GFP-GluN2C-M1stop subunit co-localized closely with an ER marker, but not a Golgi apparatus (GA) marker, supporting the notion that these constructs are not targeted to a different subcellular compartment such as the lysosomes (Figure S3).

**FIGURE 3:**
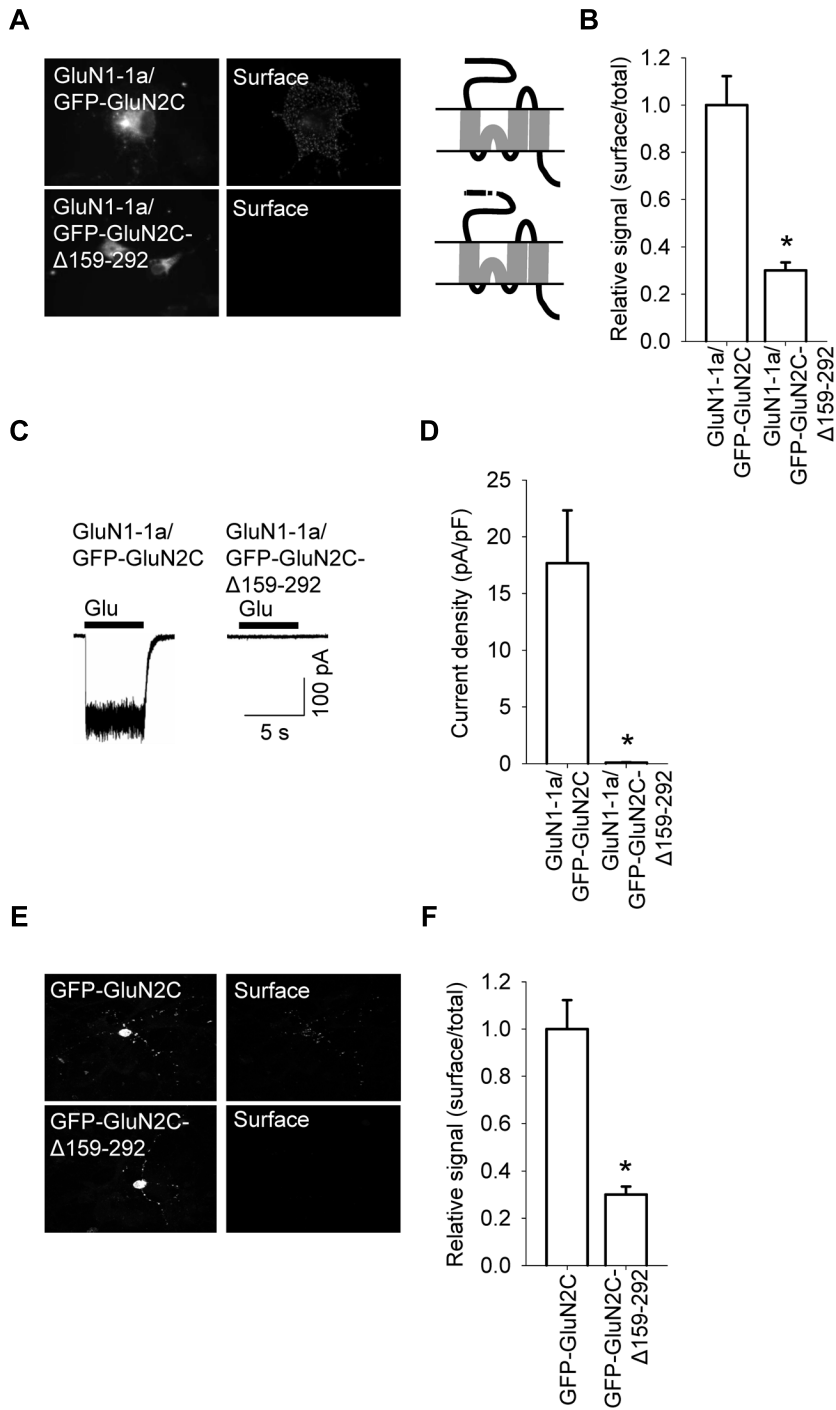
**A specific region within the N-terminus of GluN2C is essential for the surface delivery of NMDA receptors. (A)** Schematic drawings of the membrane topology of the indicated full-length GluN2 subunits and representative images of total (left panel) and surface (right panel) NMDA receptor pools in transfected COS-7 cells. **(B)** Summary of the normalized ratios of surface and total expression of the indicated NMDA receptor subunits measured using fluorescence microscopy. *p < 0.05 (relative to GluN1-1a/GFP-GluN2C); ANOVA. **(C)** Whole-cell voltage-clamp recordings were performed in HEK293 cells expressing the indicated NMDA receptor subunit combinations. Currents were elicited by applying a 5-sec pulse of 1 mM glutamate (indicated by the filled bar); representative traces are shown. **(D)** Quantitative analysis of peak current density (pA/pF) mediated by the indicated NMDA receptors; *n* ≥ 21. *p < 0.05 (relative to GluN1-1a/GFP-GluN2C); Student’s *t*-test. **(E)** Representative images of total (left panel) and surface (right panel) NMDA receptor pools in CGCs. **(F)** Summary of the normalized ratios of surface and total expression of NMDA receptors measured using confocal microscopy. *p < 0.05 (relative to GFP-GluN2C); Student’s *t*-test.

A previous study also found that deleting the A2 segment reduces the surface expression of GluN1/GluN2A receptors (Qiu et al., 2009). Therefore, we deleted the A2 segment from the GluN2C subunit (GFP-GluN2C-Δ159–292). Expressing this construct together with the GluN1-1a subunit in COS-7 cells significantly reduced the surface expression of the receptors (**Figures 3A,B**). Next, we used electrophysiology to confirm these microscopy findings. We performed whole-cell voltage-clamp recordings of HEK293 cells expressing GluN1-1a/GFP-GluN2C receptors or GluN1-1a/GFP-GluN2C-Δ159–292 receptors. Applying 1 mM glutamate (for 5 sec at a membrane potential of -60 mV) elicited receptor-mediated currents in cells expressing GluN1/GluN2C channels, and these currents were significantly smaller in cells expressing the GluN2C-Δ159–292 subunit (**Figures 3C,D**). To identify the intracellular compartment to which the mutant GluN1-1a/GFP-GluN2C-Δ159–292 receptors trafficked (given that they were not present at the cell surface), we performed immunofluorescence experiments using COS-7 cells that expressed GluN1-1a/GluN2C or GluN1-1a/GluN2C-Δ159–292 receptors and co-stained the cells with antibodies against ER and GA markers. These experiments revealed that these receptor combinations (including wild-type receptors) clearly co-localized with the ER, but not with the GA (Figures S4 and S5). Finally, we overexpressed the GFP-GluN2C and GFP-GluN2C-Δ159–292 subunits in cultured CGCs and compared their surface delivery using confocal microscopy. We found that the GFP-GluN2C-Δ159–292 subunit was significantly reduced at the cell surface (**Figures 3E,F**). In all cases, the total expression of the mutant GluN2C subunit did not differ significantly from the corresponding control (i.e., full-length) GluN2C subunit. Based on previously published results obtained from GluN1/GluN2A receptors (Qiu et al., 2009), we hypothesized that although the structural differences within the N-terminal regions do not likely account for the observed differences in surface expression between the various GluN2-containing receptors, the N-terminus clearly plays an important role in delivering GluN1/GluN2C receptors to the cell surface.

**FIGURE 4:**
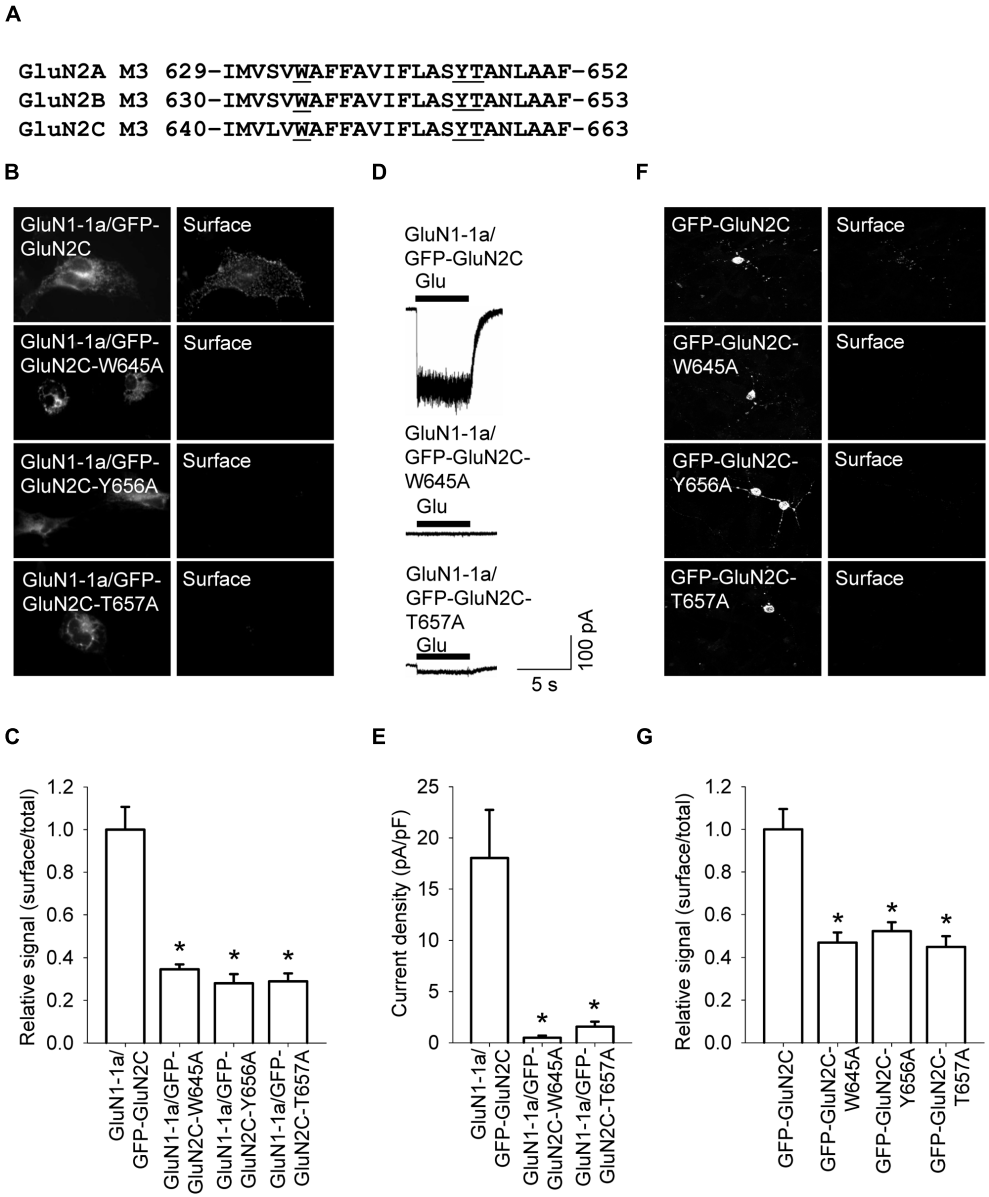
**Three amino acid residues within the M3 domain of the GluN2C subunit are essential for delivering full-length NMDA receptors to the cell surface. (A)** The sequences of the M3 domains of the GluN2A, GluN2B, and GluN2C subunits are shown; the three residues that were replaced with alanine residues are underlined. **(B)** Representative images of total (left panel) and surface (right panel) pools of NMDA receptors expressed in COS-7 cells. **(C)** Summary of the normalized ratios of surface and total expression of the indicated NMDA receptor subunits measured using fluorescence microscopy. *p < 0.05 (relative to GluN1-1a/GFP-GluN2C); ANOVA. **(D)** Whole-cell voltage-clamp recordings were performed in HEK293 cells expressing the indicated NMDA receptor subunits. Currents were elicited by applying a 5-sec pulse of 1 mM glutamate (indicated by the filled bar); representative traces are shown. **(E)** Quantitative analysis of peak current density (pA/pF) mediated by the indicated NMDA receptors; *n* ≥ 21. *p < 0.05 (relative to GluN1-1a/GFP-GluN2C); ANOVA. **(F)** Representative images of total (left panel) and surface (right panel) GluN2 pools in CGCs. **(G)** Summary of the normalized ratios of surface and total expression of NMDA receptor subunits measured using confocal microscopy. *p < 0.05 (relative to GFP-GluN2C); ANOVA.

We recently reported that specific residues within the M3 domains of both the GluN2A and GluN2B subunits are essential for delivering functional NMDA receptors to the cell surface (Kaniakova et al., 2012a). Based on this finding, we asked whether the M3 domain in GluN2C plays a similar role in the delivery of NMDA receptors to the cell surface. We therefore generated three constructs in which the amino acid residue at position 645, 656, or 657 (within the M3 domain) was replaced with an alanine residue (yielding constructs GFP-GluN2C-W645A, GFP-GluN2C-Y656A, and GFP-GluN2C-T657A, respectively); these residues are homologous to previously identified key residues in the GluN2A and GluN2B receptors (Kaniakova et al., 2012a; **Figure 4A**). When co-expressed with the GluN1-1a subunit in COS-7 cells, each mutant GluN2C subunit had reduced surface expression compared to wild-type GluN2C (**Figures 4B,C**). Consistent with these results, HEK293 cells expressing GluN1-1a/GFP-GluN2C-W645A or GluN1-1a/GFP-GluN2C-T657A receptors had reduced glutamate-induced currents (**Figures 4D,E**); similar results were obtained using cultured CGCs transfected with GFP-GluN2C-W645A, GFP-GluN2C-Y656A, or GFP-GluN2C-T657A subunits (**Figures 4F,G**). Together, these results support our conclusion that the M3 domain in GluN2C is essential for delivering the receptor to the cell surface. Lastly, co-localization experiments revealed that GluN1-1a/GFP-GluN2C-W645A and GluN1-1a/GFP-GluN2C-T657A receptors are present mostly in the ER (Figures S4 and S5).

Finally, we asked whether structural differences in the M3 domains among the various GluN2 subunits can explain the observed differences in surface expression. Because the M3 domains of GluN2A and GluN2C differ by only one amino acid residue (Figure S6), we generated a full-length GluN2A subunit containing the GluN2C M3 domain (GFP-GluN2A-S632L) and a full-length GluN2C subunit containing the GluN2A M3 domain (GFP-GluN2C-L634S). When co-expressed with GluN1-1a subunits in COS-7 cells, these mutant subunits did not differ significantly from their respective controls in terms of surface expression (Figure S6). Thus, these data suggest that although the M3 domain in GluN2C is essential for delivering GluN2C-containing NMDA receptors to the cell surface, other regions in the GluN2C subunit are likely responsible for the differences in surface delivery of GluN2A-, GluN2B-, and GluN2C-containing receptors.

**FIGURE 5:**
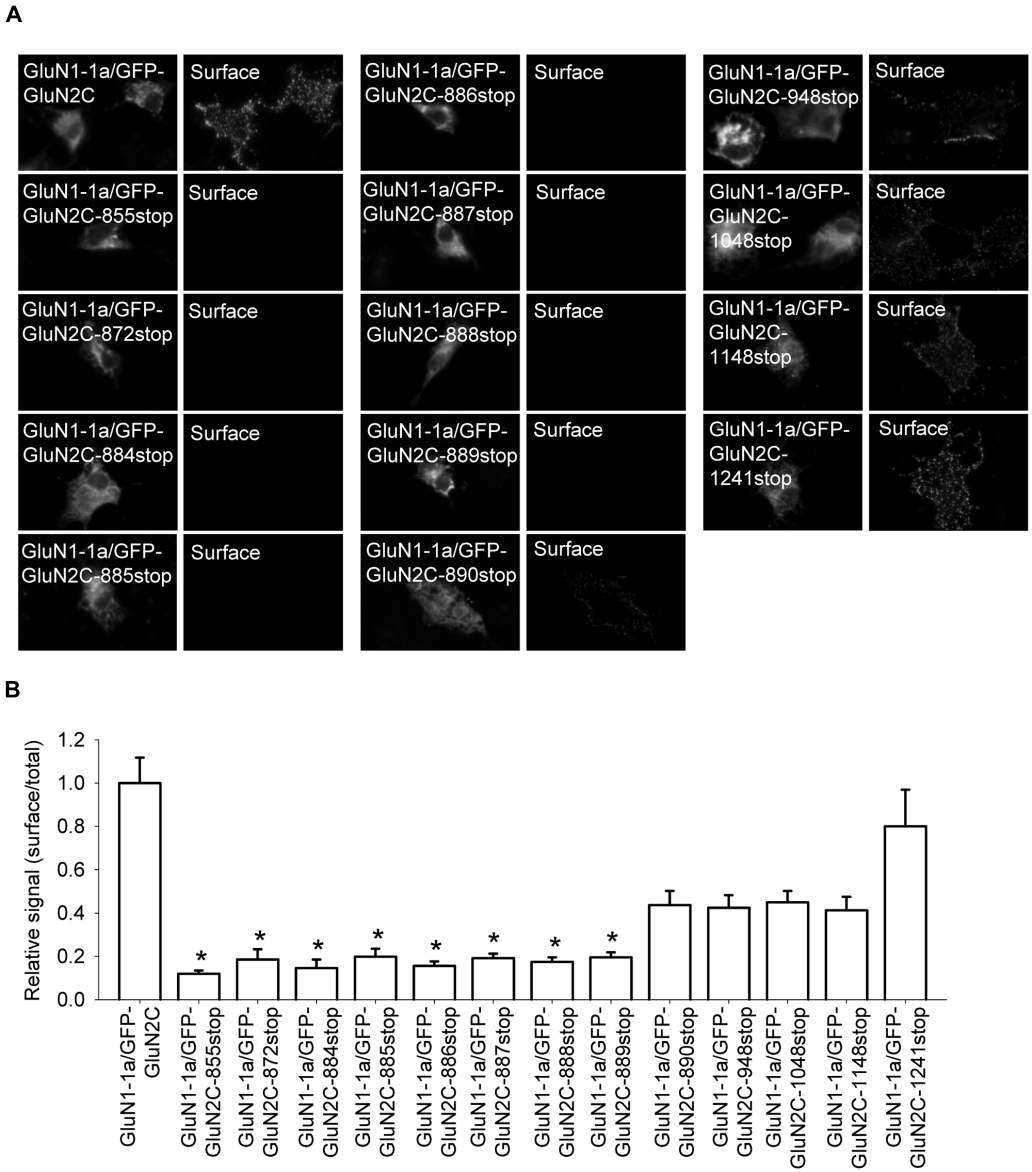
**The intracellular C-terminal domain of GluN2C is essential for the surface delivery of NMDA receptors. (A)** Representative images of total (left panel) and surface (right panel) pools of labeled NMDA receptor subunits expressed in COS-7 cells. **(B)** Summary of the normalized ratios of surface and total expression of NMDA receptors measured using fluorescence microscopy. *p < 0.05 (relative to GluN1-1a/GFP-GluN2C); ANOVA.

**FIGURE 6:**
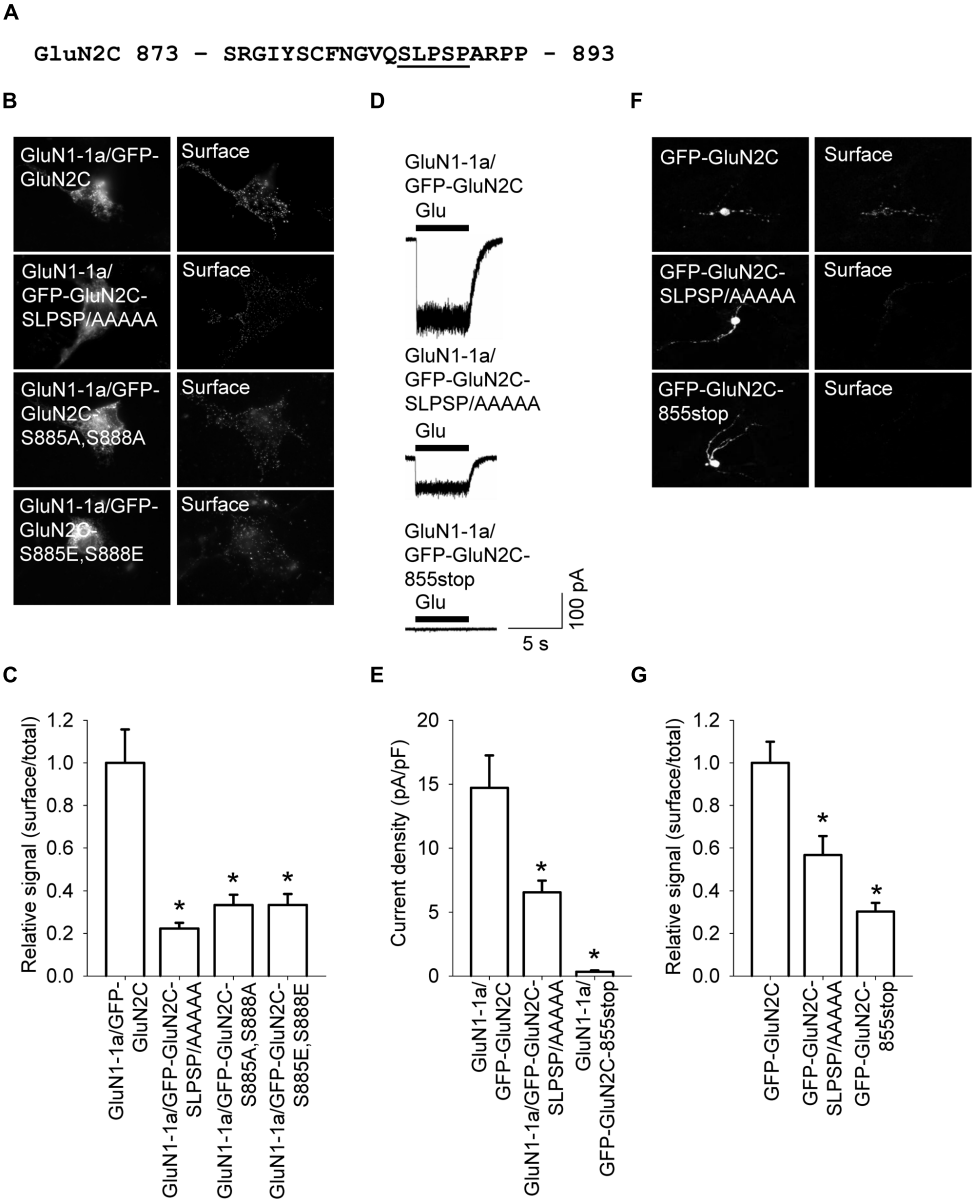
**A short amino acid sequence in the proximal C-terminus of GluN2C is essential for surface delivery of NMDA receptors. (A)** The amino acid sequence of the proximal part of the C-terminus of GluN2C; the residues that were replaced with alanines are underlined. **(B)** Representative images of total (left panel) and surface (right panel) pools of NMDA receptors expressed in COS-7 cells.** (C)** Summary of the normalized ratios of surface and total expression of the indicated NMDA receptor subunits measured using fluorescence microscopy. *p < 0.05 (relative to GluN1-1a/GFP-GluN2C); ANOVA. **(D)** Whole-cell voltage-clamp recordings were performed in HEK293 cells expressing the indicated NMDA receptor subunits. Currents were elicited by applying a 5-sec pulse of 1 mM glutamate (indicated by the filled bar); representative traces are shown. **(E)** Quantitative analysis of the peak current density (pA/pF) mediated by the indicated NMDA receptors; *n* ≥ 19. *p < 0.05 (relative to GluN1-1a/GFP-GluN2C); ANOVA. **(F)** Representative images of total (left panel) and surface (right panel) GluN2 pools in CGCs. **(G)** Summary of the normalized ratios of surface and total expression of NMDA receptor subunits measured using confocal microscopy. *p < 0.05 (relative to GFP-GluN2C); ANOVA.

The C-terminal region of GluN subunits was previously implicated in regulating the delivery of NMDA receptors to the cell surface (Traynelis et al., 2010; Sanz-Clemente et al., 2012). To determine whether the C-terminus of the GluN2C subunit regulates the surface delivery of GluN2C-containing NMDA receptors, we first generated a GluN2C subunit that lacks the C-terminal domain (GFP-GluN2C-855stop); the protein was truncated at a similar position as the truncated versions of GluN2A and GluN2B in previous studies (Vissel et al., 2001; Horak et al., 2008). Interestingly, when co-expressed in COS-7 cells with the GluN1-1a subunit, receptors containing the truncated GluN2C failed to traffic to the cell surface (**Figures 5A,B**). Therefore, we generated a series of C-terminal truncated GluN2C subunits in order to determine whether a specific structural element is essential for the forward trafficking of GluN2C-containing NMDA receptors. We found that the surface expression of seven truncated versions of the GluN2C subunit (truncated from residue 872 through residue 889) is significantly reduced; in contrast, five truncated GluN2C subunits (truncated from residue 890 through residue 1241) had normal levels of surface expression (**Figures 5A,B**). These results suggest that the region adjacent to residue 889 is critically involved in regulating the surface expression of GluN2C-containing receptors. To test this idea, we generated a full-length pentamutant GFP-GluN2C subunit in which the SLPSP sequence (amino acid residues 885–889) was replaced with alanines, yielding GFP-GluN2C-SLPSP/AAAAA (**Figure 6A**). We then co-expressed this construct together with GluN1-1a and used immunofluorescence to measure surface NMDA receptors. The GluN1-1a/GFP-GluN2C-SLPSP/AAAAA receptors were delivered to the cell surface at significantly lower levels than control receptors (**Figures 6B,C**). Similar results were obtained when the GluN1-1a and GFP-GluN2C-SLPSP/AAAAA subunits were expressed in HEK293 cells (**Figures 6D,E**) or cultured CGCs (**Figures 6F,G**); however, in both expression systems, the GFP-GluN2C-855stop subunit caused even less surface expression (**Figures 6D–G**).

Because the SLPSP motif contains two serine residues, we asked whether surface delivery of the GluN2C-containing receptors is regulated by phosphorylation at these sites. We therefore generated two mutant GluN2C constructs; one construct has alanines substituted for both serines (GFP-GluN2C-S885A,S888A), and the other construct has both serines replaced with the phosphomimetic residue glutamate (GFP-GluN2C-S885E,S888E). We then expressed these mutant subunits together with GluN1-1a in COS-7 cells and measured the surface expression of the receptors. We found that both GluN1-1a/GFP-GluN2C-S885A,S888A and GluN1-1a/GFP-GluN2C-S885E,S888E receptors had reduced surface expression (**Figures 6B,C**). Taken together with our co-localization studies using GluN1-1a/GFP-GluN2C-855stop receptors (Figures S4 and S5), we propose that the proximal C-terminus of the GluN2C subunit—and the SLPSP motif in particular—is a critical structural element that regulates the surface delivery of GluN1/GluN2C receptors (see also Discussion). We also suggest that the C-terminus of GluN2C is the most likely structural element underlying the decreased surface expression of GluN1/GluN2C receptors compared to GluN1/GluN2A and GluN1/GluN2B receptors.

## DISCUSSION

The early processing and intracellular transport of NMDA receptors to the cell surface is regulated by specific mechanisms that ensure that only properly assembled receptors containing the appropriate subunits are released from the ER and delivered to the cell surface. Here, we investigated the mechanism by which the GluN2C subunit regulates the surface delivery of NMDA receptors. Using a combination of molecular biology, microscopy, and electrophysiology in mammalian cell lines and CGCs expressing recombinant NMDA receptors, we found that the delivery of GluN1/GluN2C receptors to the cell surface is reduced considerably compared to both GluN1/GluN2A and GluN1/GluN2B receptors. Furthermore, we identified three regions within different domains of the GluN2C subunit that play a key role in the surface expression of GluN2C-containing NMDA receptors. We conclude that the GluN2C subunit regulates the forward trafficking of NMDA receptors by a unique mechanism that differs from other NMDA receptor types.

### ROLE OF THE GluN2C SUBUNIT IN THE FORWARD TRAFFICKING OF NMDA RECEPTORS

Our finding that GluN1/GluN2A and GluN1/GluN2B receptors are differentially targeted to the cell surface of mammalian cell lines is consistent with previously published results (Chen et al., 1999). However, in cultured CGCs, we found no difference in surface expression between GluN1/GluN2A and GluN1/GluN2B receptors. This discrepancy may be due to the presence of endogenous NMDA receptor subunits in cultured neurons; these endogenous subunits can form multiple NMDA receptor complexes, including triheteromeric receptors (Hansen et al., 2014). The finding that surface targeting of GluN2C-containing receptors is reduced compared to GluN2A- and GluN2B-containing receptors—which was observed in both mammalian cell lines and cultured CGCs—indicates that the GluN2C subunit contains critical structural elements that control the trafficking of GluN2C-containing receptors. These GluN2C-specific elements are likely recognized by specific protein-protein binding partners, including sorting nexin 27 (SNX27) and 14-3-3-epsilon (Chen and Roche, 2009; Cai et al., 2011), as well as other unidentified proteins. Indeed, when expressed alone (i.e., without GluN1), the GluN2C subunit was retained in the intracellular compartment, as shown previously for GluN2A and GluN2B (Mcllhinney et al., 1998; Horak et al., 2008). This finding suggests that co-assembly of the GluN1 subunit in the receptor is essential for the release of all GluN2 subunit types from the ER.

### ROLE OF DISTINCT REGIONS IN THE GluN2C SUBUNIT IMPLICATED IN THE FORWARD TRAFFICKING of NMDA RECEPTORS

We identified three distinct regions within the GluN2C subunit that are essential for driving the surface delivery of GluN1/GluN2C receptors. First, truncating the GluN2C subunit immediately downstream of the M1 domain caused the protein to be retained in the intracellular compartment, and this retention was released by deleting the N-terminal A2 segment. However, deleting the N-terminal A2 segment from the full-length (i.e., non-truncated) GluN2C subunit reduced the surface expression of GluN1/GluN2C receptors. Interestingly, a similar phenomenon was reported previously for GluN2A, but not GluN2B (Qiu et al., 2009). Given that the A2 segment is relatively well conserved among GluN2 subunits, it is currently unclear why the A2 segment regulates GluN2A and GluN2C differently than GluN2B. It is possible that the A2 segment of some GluN2 subunits can interact with specific binding partner(s); alternatively, the presence of a specific A2 segment is required to enable the GluN1/GluN2 heterotetramer to pass the intracellular quality control checkpoints (with the ER serving as the most likely checkpoint). The latter possibility may be supported by the finding that the GluN1 homodimer must dissociated in order to form the GluN1/GluN2 heterotetramer (Farina et al., 2011).

Second, our experiments revealed the identity of three amino acid residues within the M3 domain of GluN2C—specifically, W645, Y656, and T657—that are important for delivering GluN2C-containing NMDA receptors to the cell surface. We previously reported that the presence of identical residues within the GluN1, GluN2A, and GluN2B subunits is essential for surface delivery of the NMDA receptors; therefore, a shared mechanism likely underlies the M3 domain’s ability to regulate the surface expression of NMDA receptors (Kaniakova et al., 2012a). In contrast to previous data with GluN1, GluN2A, and GluN2B subunits with mutations in the M3 domain, we observed extremely small currents in GluN1/GluN2C receptors with a mutation in the GluN2C subunit’s M3 domain. One explanation may be that the reduced surface expression of wild-type GluN2C-containing receptors—compared to GluN1/GluN2A and GluN1/GluN2B receptors—is further reduced by the same mechanisms, as in the case of GluN1/GluN2A and GluN1/GluN2B receptors. Alternatively, the GluN1/GluN2C receptors may be regulated via their membrane domains more tightly than other receptor types, and this may be reflected in their reduced surface localization. The membrane domains of GluN subunits were found to be essential for regulating NMDA receptors (Ren et al., 2007; Salous et al., 2009). Moreover, the presence of the M4 domain in GluN1 and GluN2 was found to be essential for forming functional receptors (Schorge and Colquhoun, 2003). Thus, based on these previous data and our observations, specific inter-membrane domain interactions are clearly essential for mediating the delivery of NMDA receptors to the cell surface; however, it is currently not clear whether their effect is mediated during ER processing and/or downstream intracellular transport (Greger et al., 2003; Cao et al., 2011; Salussolia et al., 2011; Kaniakova et al., 2012b).

Finally, we found that deleting the entire C-terminus of GluN2C significantly reduces the number of functional NMDA receptors at the cell surface. Interestingly, deleting the C-terminus of GluN2A or GluN2B does not have such a profound effect (Vissel et al., 2001; Horak et al., 2008). Our series of deletions and mutations revealed a critical structural element within the proximal C-terminal region of the GluN2C subunit; this 5-residue motif (SLPSP) regulates the surface expression of GluN1/GluN2C receptors. Whether the SLPSP motif interacts with a specific binding partner remains unclear. Indeed, we cannot exclude the possibility that additional structural elements within the C-terminus of GluN2C—aside from the SLPSP motif—also regulate the transport of GluN1/GluN2C receptors. This view is supported by a compelling study that identified the RHASLP motif in the C-terminus of GluN2C as a 14-3-3 binding motif (Chen and Roche, 2009). Moreover, we found that deleting the PDZ-binding motif in the GluN2C subunit (i.e., our GluN2C-1241stop construct) did not affect the surface expression of NMDA receptors, which is consistent with a previous study that found that phosphorylation of the serine residues adjacent to the PDZ-binding motif does not regulate the trafficking of GluN1/GluN2C receptors (Chen et al., 2006). Based on this large body of data, we propose that the C-terminus of GluN2C subunits—including the SLPSP motif—plays a unique and specific role in regulating the delivery of GluN1/GluN2C receptors to the cell surface. At this time, our data cannot be used to determine whether the GluN2C C-terminus—including the SLPSP motif—plays a role in ER processing and/or intracellular transport of NMDA receptors. Nevertheless, the C-terminus of GluN2C has relatively low homology with the C-termini of GluN2A and GluN2B (Ishii et al., 1993); therefore, the SLPSP motif within the C-terminus of GluN2C does not have a corresponding motif (i.e., located the same distance from the M4 domain) in the GluN2A and GluN2B subunits.

### PHYSIOLOGICAL IMPLICATIONS

Our results clearly demonstrate that multiple structural elements within the GluN2C subunit regulate the transport of GluN2C-containing NMDA receptors. Although it is currently not clear why cells use multiple mechanisms to regulate the trafficking of various NMDA receptor types, it is possible that this strategy ensures that only properly folded GluN2C-containing receptors are transported to the cell surface. Indeed, specific regulatory mechanisms may be used under specific circumstances (e.g., during the activity-driven stimulation of synapses); thus, having several regulatory options available enables the cell to react appropriately under different conditions. Interestingly, mice that express a GluN2C subunit that lack the C-terminus have clear deficits in motor coordination (Sprengel et al., 1998); this observation is consistent with our finding that the C-terminus of GluN2C is an essential element for delivering NMDA receptors to the cell surface. Given that the proper regulation of NMDA receptors is essential for many processes, including excitatory neurotransmission, synaptic plasticity, learning, and memory consolidation, our results provide key insight into the molecular mechanisms that underlie the function of NMDA receptors. These results may also facilitate the development of new therapeutic strategies for treating a wide variety of diseases that are associated with aberrant NMDA receptor trafficking.

## AUTHOR CONTRIBUTIONS

Martin Horak, Katarina Lichnerova, Kristyna Skrenkova, Ladislav Vyklicky, and Martina Kaniakova performed the experiments, Martin Horak, Kristyna Skrenkova, Ladislav Vyklicky, and Martina Kaniakova analyzed the data, and Martin Horak wrote the manuscript with input from the co-authors. All authors read and approved the final version of the manuscript.

## Conflict of Interest Statement

The authors declare that the research was conducted in the absence of any commercial or financial relationships that could be construed as a potential conflict of interest.
