# Supplementary material for: Distinct regions within the GluN2C subunit regulate the surface delivery of NMDA receptors
Source: Front Cell Neurosci. 2014 Nov 10;8:375. doi: 10.3389/fncel.2014.00375 (PMC4226150; doi:10.3389/fncel.2014.00375)
Supplement: Supplementary file 1 [file Data_Sheet_1.DOC]

**Distinct regions within the GluN2C subunit regulate the surface delivery of NMDA receptors**

**Supplementary material**

**Figure legends**

**Figure S1. The difference in the surface delivery of the YFP-GluN1-1a/GluN2A and YFP-GluN1-1a/GluN2C receptors. (A)** Representative images of the total (left panel) and surface (right panel) pools of GluN1/GluN2 receptors expressed in COS-7 cells. **(B)** Summary of the normalized intensity ratios of surface and total NMDA receptors expressed in COS-7 cells and visualized using immunofluorescence. *, p < 0.05 (relative to YFP-GluN1-1a/GluN2A); Student’s t-test.

**Figure S2. The GluN2 subunits are retained intracellularly without the presence of the GluN1 subunit. (A)** Representative images of the total (left panel) and surface (right panel) pools of GluN subunits expressed in COS-7 cells. **(B)** Summary of the normalized intensity ratios of surface and total NMDA receptors expressed in COS-7 cells and visualized using immunofluorescence. *, p < 0.05 (relative to GluN1-1a/GFP-GluN2A); ANOVA.

**Figure S3. Subcellular localisation of the truncated GluN2 subunits.** The distribution of the GFP- GluN2A-M1stop and GluN2C-M1stop subunits closely matches the distribution of an ER marker PDI **(A)** but not a GA marker 58K **(B)** Images were taken on fixed transfected COS-7 cells using a confocal microscope. Scale bar, 20 µm.

**Figure S4. Subcellular localisation of the indicated GluN1/GluN2C receptors.** The distribution of the indicated NMDA receptors closely matches the distribution of an ER marker PDI. Images were taken on fixed transfected COS-7 cells using a confocal microscope.

**Figure S5. Subcellular localisation of the indicated GluN1/GluN2C receptors.** The distribution of the indicated NMDA receptors closely does not match the distribution of a GA marker 58K. Images were taken on fixed transfected COS-7 cells using a confocal microscope.

**Figure S6. The surface expression of chimeric NMDA receptors. (A)** The sequences of the M3 domains of the GluN2A, GluN2B, and GluN2C subunits are shown; the single amino acid residues that differ between the GluN2A, GluN2B, and GluN2C subunits are boxed. **(B)** Representative images of total (left panel) and surface (right panel) pools of NMDA receptors expressed in COS-7 cells. **(C)** Summary of the normalized ratios of surface and total expression of the indicated NMDA receptor subunits measured using fluorescence microscopy. p > 0.05 (relative to GluN1-1a/GFP-GluN2A or GluN1-1a/GFP-GluN2C); Student’s t-test.

**Supplementary methods**

Microscopy

For co-localization studies, COS-7 cells were washed, fixed in 4% PFA in PBS, permeabilized by 0.25% TX-100 in PBS (w/v) for 5 min and labeled with primary rabbit anti-GFP (Millipore; 1:1000) and primary mouse anti-PDI (Abcam, Cambridge, UK, 1:200; ER marker) or mouse anti-58K Golgi protein (Abcam, 1:200; GA marker) and secondary goat anti-mouse Alexa Fluor 647 and anti-rabbit Alexa Fluor 488 (Invitrogen) antibodies. Cells were then mounted with ProLong Antifade reagent (Invitrogen). The images were taken on Leica SPE confocal microscope and processed using ImageJ software (NIH).
