# Supplementary figures and images for: Distinct regions within the GluN2C subunit regulate the surface delivery of NMDA receptors
Source: Front Cell Neurosci. 2014 Nov 10;8:375. doi: 10.3389/fncel.2014.00375 (PMC4226150; doi:10.3389/fncel.2014.00375)

**Figure S1**

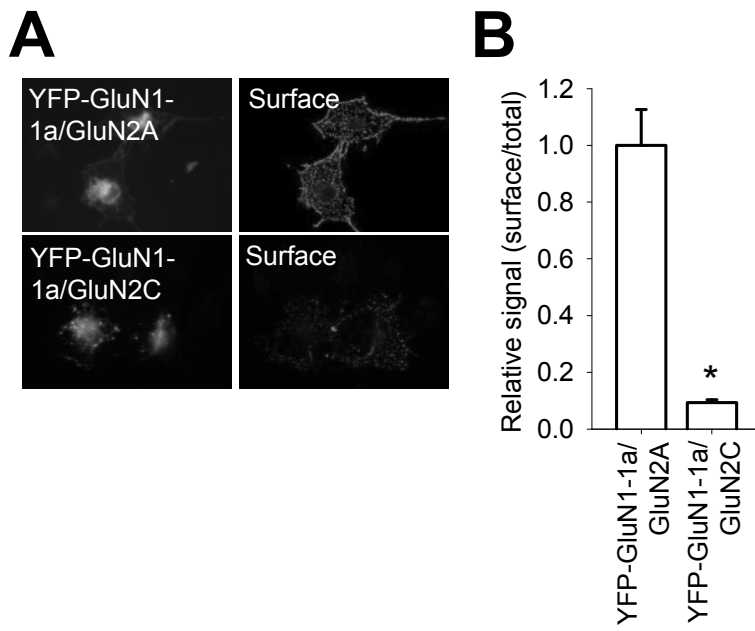

Supplement: Supplementary file 2 [file Image_1.PDF]

**Figure S2**

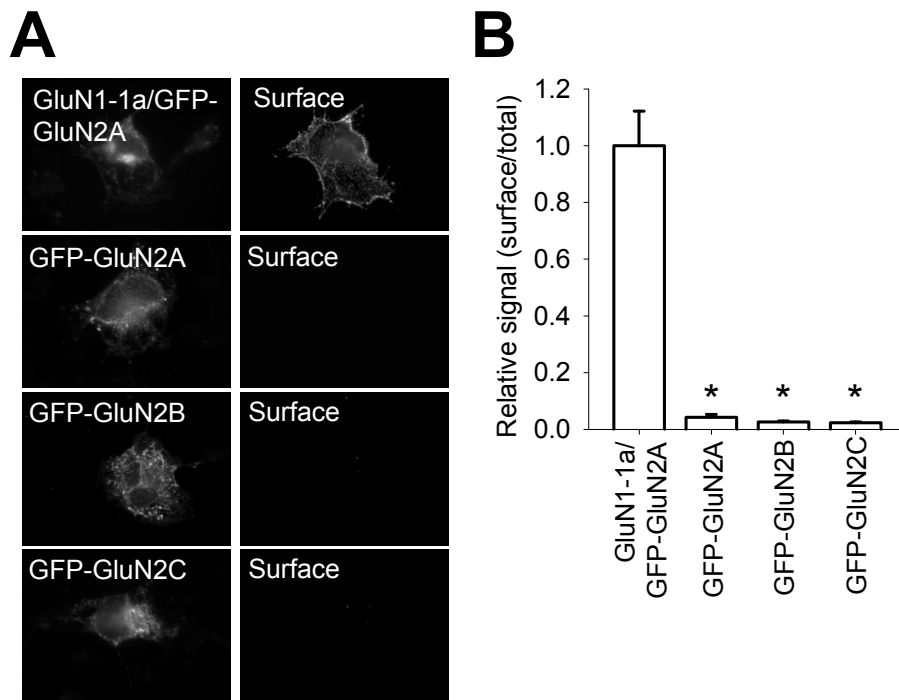

Supplement: Supplementary file 3 [file Image_2.PDF]

**Figure S3**

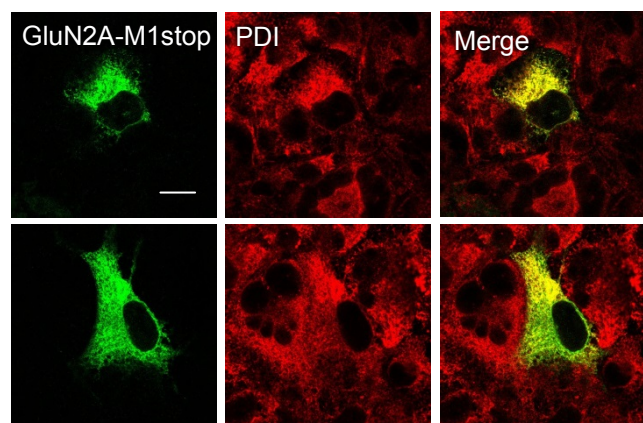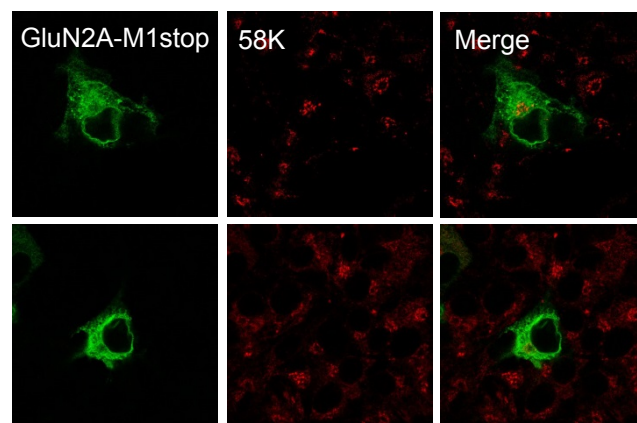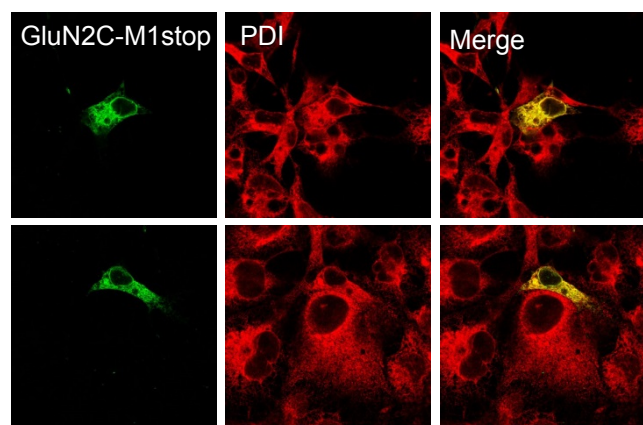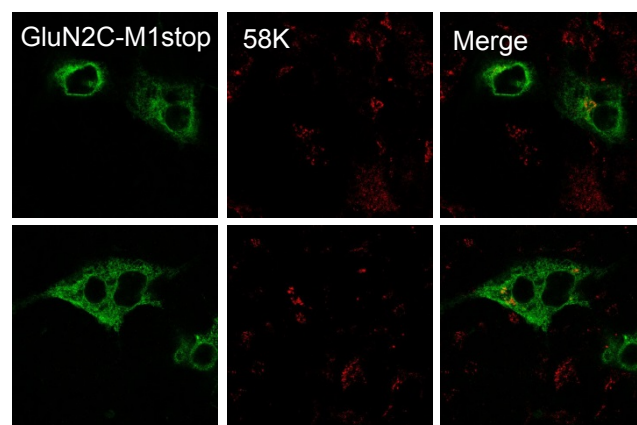

Supplement: Supplementary file 4 [file Image_3.PDF]

**Figure S4**

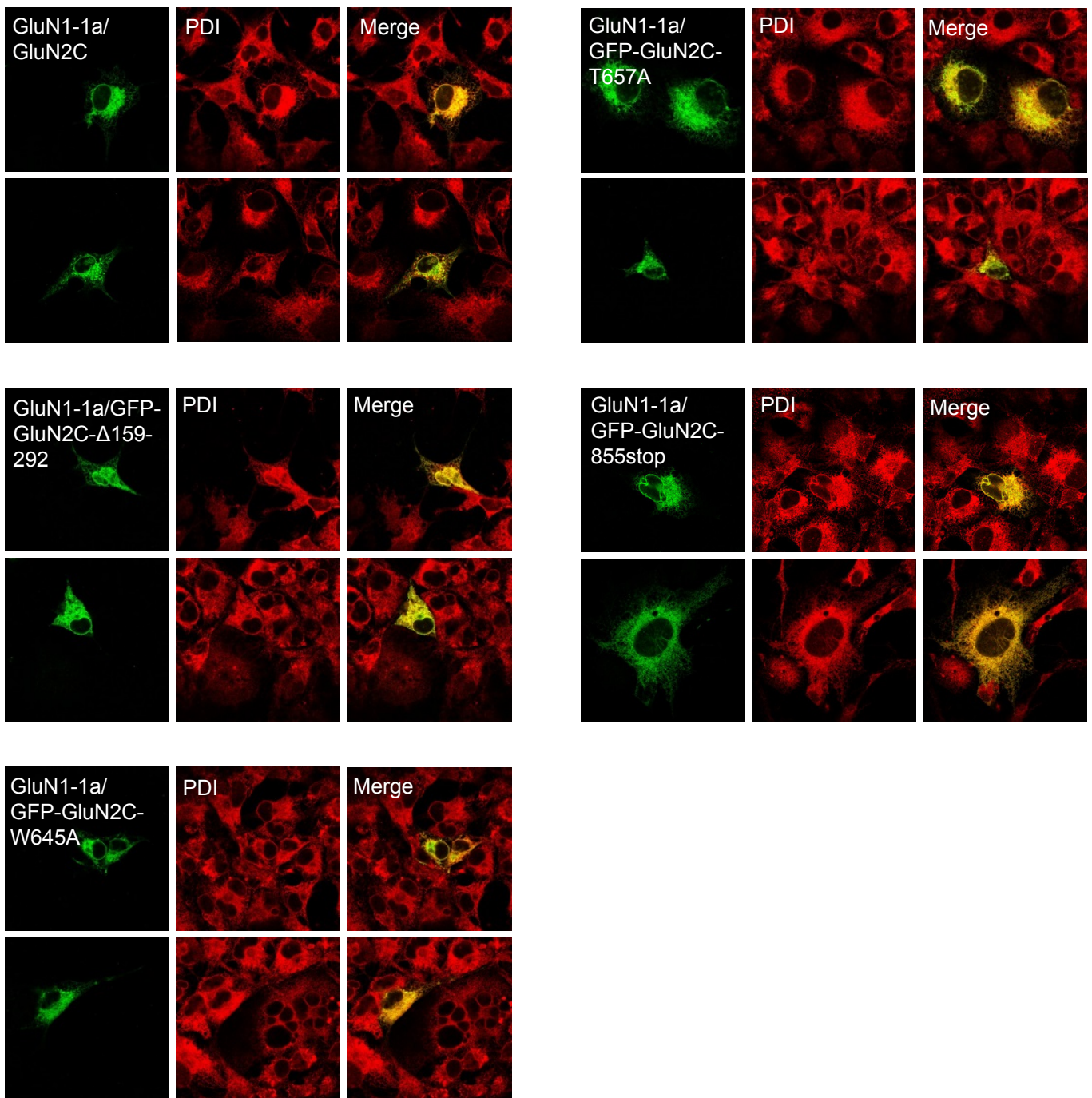

Supplement: Supplementary file 5 [file Image_4.PDF]

**Figure S5**

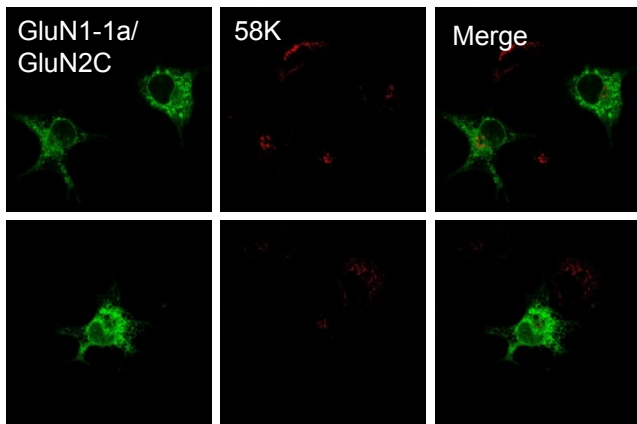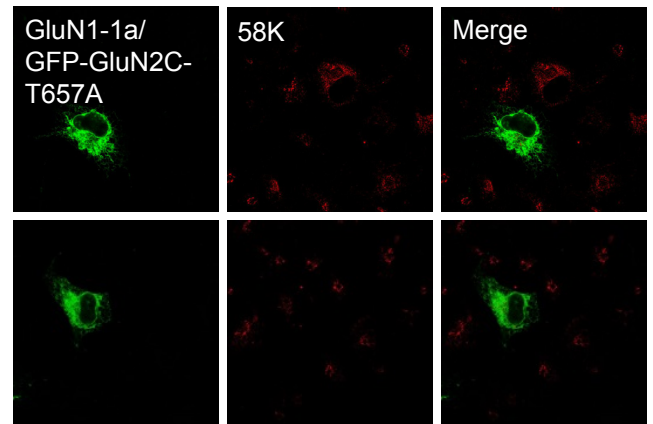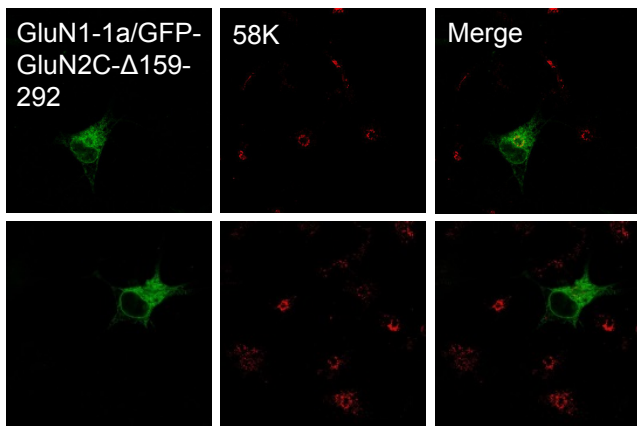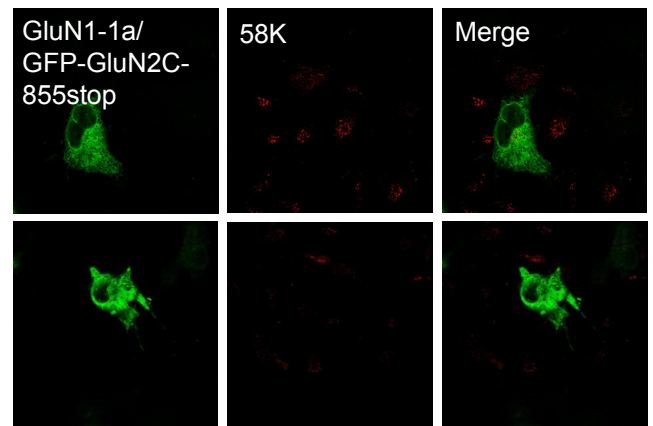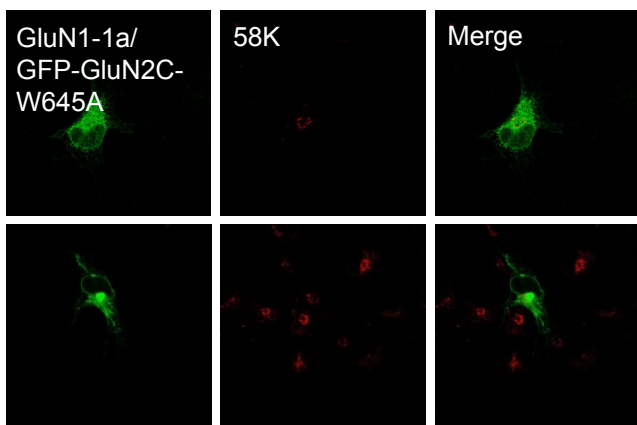

Supplement: Supplementary file 6 [file Image_5.PDF]
