# Supplementary material for: Distinct regions within the GluN2C subunit regulate the surface delivery of NMDA receptors
Source: Front Cell Neurosci. 2014 Nov 10;8:375. doi: 10.3389/fncel.2014.00375 (PMC4226150; doi:10.3389/fncel.2014.00375)

A

Figure S6

```
GluN2A M3 629-IMVSVWAFFAVIFLASYTANLAAF-652
GluN2B M3 630-IMVSVWAFFAVIFLASYTANLAAF-653
GluN2C M3 640-IMVLVWAFFAVIFLASYTANLAAF-663
```

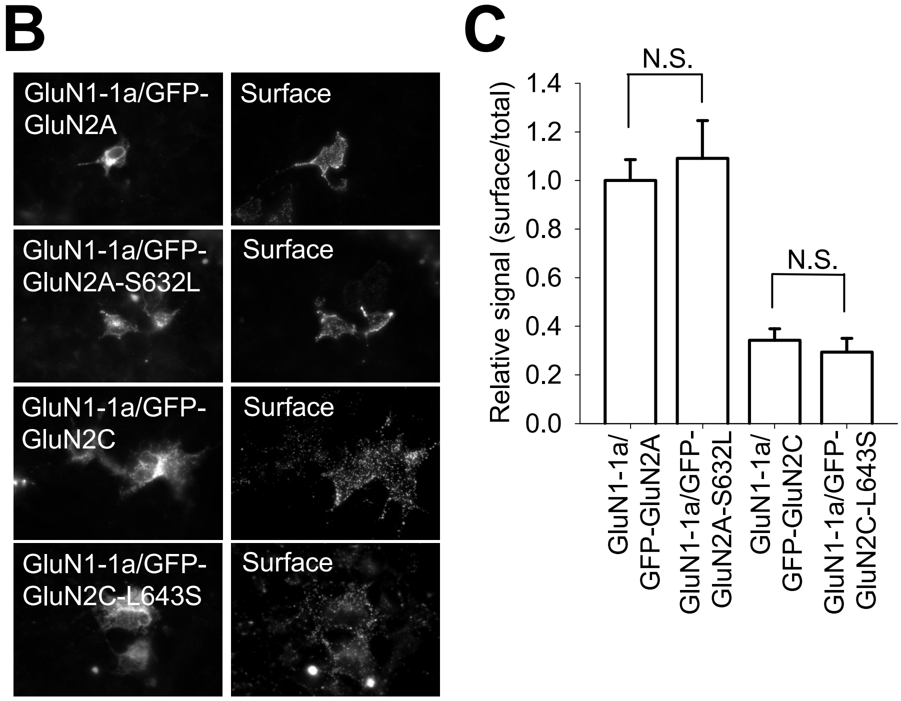

Supplement: Supplementary file 7 [file Image_6.PDF]
